# Knowledge hiding and social exchange theory: a systematic review and meta-analysis

**DOI:** 10.3389/fpsyg.2024.1516815

**Published:** 2025-01-06

**Authors:** Zijun Zhang, Yoshi Takahashi, Roksana Binte Rezwan

**Affiliations:** ^1^Graduate School of Humanities and Social Sciences, Hiroshima University, Hiroshima, Japan; ^2^Institute for International Strategy, Tokyo International University, Tokyo, Japan

**Keywords:** social exchange theory, norms of reciprocity, knowledge management, knowledge hiding, meta-analysis, literature review

## Abstract

The literature on the antecedents and consequences of knowledge hiding remains fragmented, limiting its practical applications. Social exchange theory (SET), one of the most widely adopted sociological frameworks, offers unique insights into the dynamics of knowledge hiding. This study synthesizes the application of SET in analyzing the nomological framework of knowledge hiding through a systematic literature review and meta-analysis. A meta-analysis was conducted based on the random-effects model and the meta-analytic structural equation modeling method, incorporating 66 primary studies with a total of 20,603 participants. Additionally, we examined the mediating role of knowledge hiding by linking key antecedents and consequences. Moreover, an exploratory analysis was conducted to investigate the moderating effects of national culture and research methodology, providing evidence to justify the true heterogeneity in the pairwise relationships between knowledge hiding and its antecedents. The research results generally support most pairwise relationships between knowledge hiding and its correlates, which were theoretically developed based on SET. This study is the first attempt to explore the explanatory power of SET in analyzing the knowledge-hiding phenomenon, and whether the establishment of a knowledge exchange loop contributes to a deeper understanding of this dyadic construct.

## 1 Introduction

In the knowledge economy, knowledge plays a key role in driving wealth growth and organizational success (Lin and Hsiao, 2014). Given the salient importance of knowledge, much of the literature focuses on how to effectively manage the intellectual capital of employees. This has led to increased investments in knowledge management technologies to validate knowledge flows and enhance information-sharing within organizations (Mårtensson, 2000). However, despite these advancements, knowledge sharing has not facilitated sufficiently to promote productivity and reinforce operational efficiency due to the emergence of knowledge hiding. Therefore, it is necessary to determine the mechanisms underlying knowledge hiding, such as how it comes into existence and how it functions. Knowledge hiding, as defined as by Connelly et al. (2012, p. 65), refers to “an intentional attempt by an individual to withhold or conceal knowledge that has been requested by one another.” Considering the counterproductive nature of knowledge hiding, it has gradually become a significant research concern in recent years, particularly in the fields of knowledge management and organizational behavior.

The literature on the antecedents and consequences of knowledge hiding is extremely fragmented. Although previous studies have presented many ways to tackle knowledge hiding from multi-faceted perspectives, their lack of generalizable conclusions has prevented the efficient transfer of these results from theory to practice (Arain et al., 2024). Moreover, the implementation of various theories provides distinctive viewpoints and conflicting results in investigating and rationalizing the knowledge-hiding phenomenon in professional settings. For example, Černe et al. (2014) suggested that knowledge hiding exerts a negative influence on individuals’ creativity because of the reciprocal distrust loop via the lens of social exchange (*β* = −0.21**). However, Zakariya and Bashir (2021) found that knowledge hiding could promote individuals’ creative performance because of feelings of envy from a social comparison perspective (β = 0.435**). Both findings seem reasonable, but we need to confirm the extent to which knowledge hiding can be examined and explained through social exchange and social comparison theories. Therefore, this meta-analysis aims to synthesize the empirical findings concerning the relationship between knowledge hiding and its correlates, while also examining the explanatory power of commonly used theories in rationalizing knowledge hiding. As social exchange theory (SET) is one of the most popular sociological theories and has been widely used in analyzing knowledge hiding, we adopt it in this meta-analysis to develop a nomological framework of knowledge hiding.

Therefore, this study makes three main contributions to the literature on knowledge hiding and fills the existing research gaps as follows. First, to address the lack of consensus regarding knowledge hiding from different theoretical backgrounds, we established a nomological framework containing a wide range of relationships with knowledge hiding based on SET (Blau, 2017). As knowledge hiding generally occurs in dyads through social interactions (Connelly et al., 2012), SET may be effective in explaining the inner nature of knowledge hiding. Second, this study also explores the intermediatory role of knowledge hiding via the meta-analytic structural equation modeling (MASEM) technique, which provides an opportunity to easily comprehend the dynamic procedures of such knowledge management failures through social exchange loops (Zhang et al., 2022). Finally, this study provides in-depth insights into the influence of moderators in justifying the variability across primary studies on the relationships between knowledge hiding and its correlates. By including national culture (collectivism and power distance) and research methodology (knowledge intensity and knowledge-hiding measures) as moderators, we provide strong explanations for the variability across studies, when the magnitude of knowledge hiding is more likely to be induced or hindered.

## 2 Social exchange theory and knowledge hiding

SET is one of the most influential paradigms used in the field of knowledge hiding and describes how much effort an individual would like to dedicate to the sustainment and development of a social relationship through a benefit–cost analysis (Blau, 2017; O’Boyle et al., 2012). Resource exchanges, such as valuable knowledge exchanges, are generally processed based on the norms of reciprocity, thus all people involved in such social interactions would expect rewards to balance or even exceed their costs. If individuals do not receive the expected amounts of returns, they intentionally hide their expertise and skills rather than share them with others to minimize potential costs created by the exchange relationship. Moreover, O’Boyle et al. (2012) suggested that individuals engage in the process of reliable exchange not only for objective goods and services, but also for intangible rewards that yield socially valued outputs such as status and admiration. Therefore, given that the benefits of social exchange are not always tangible and objective, the applicable scope of SET in justifying the formation and influencing mechanisms of knowledge hiding can be greatly extended.

**Figure 1:**
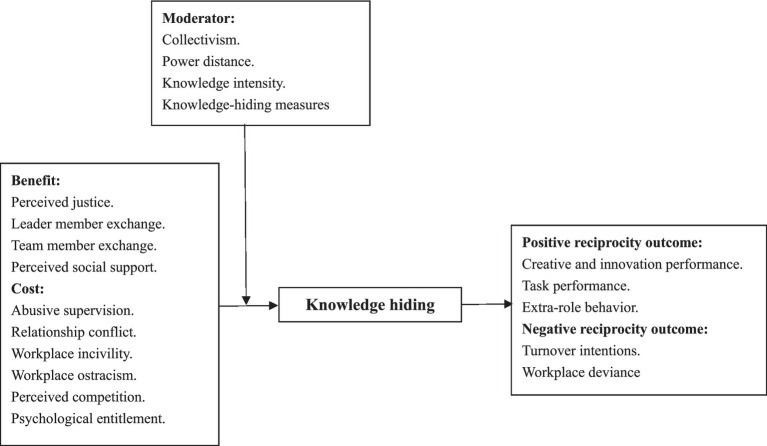
Hypothesized meta-analytic model.

The extant literature reports a series of antecedents and outcomes of knowledge hiding that have been successfully explained through the lens of social exchanges from personal, interpersonal, and organizational perspectives. Although SET has certain explanatory power in rationalizing the knowledge-hiding phenomenon, it still has some underlying shortcomings, such as the extent to which it can truly describe the procedure of knowledge hiding, which would lead to difficulties in theoretical explanations as well as in practical implementation. Accordingly, in this study, a nomological framework of knowledge hiding was established based on SET to measure the reliability and validity of the proposed pairwise relationships, as well as their theoretical effectiveness (see Figure 1).

From the lens of social exchange, we divided the antecedents of knowledge hiding into two different groups based on the benefit–cost analysis (Blau, 2017). The terms “Benefit” and “Cost” represent the motivating and inhibiting factors that impact on one’s engagement in the process of social exchange, thereby shaping individuals’ attitudes toward knowledge hiding. It is expected that antecedents in the “Benefit” category may have a negative association with knowledge hiding, while variables in the “Cost” category are anticipated to exhibit a positive relationship with knowledge hiding. Besides, the consequences of knowledge hiding were also categorized into “positive reciprocity outcome” (returning kindness to kindness) and “negative reciprocity outcome” (returning harm to harm) based on their inner nature. Individuals are likely to respond to the existence of knowledge hiding with more negative actions and less positive actions in accordance with the norms of reciprocity, as B’s reaction to A is contingent on A’s behavior to B (Gouldner, 1960). Finally, recent meta-analyses have highlighted the importance of examining moderators in the field of knowledge hiding (Arain et al., 2024; Škerlavaj et al., 2023). Therefore, in this study, we included moderators from the perspectives of national culture (collectivism and power distance) and research methodology (knowledge intensity and knowledge-hiding measures) to investigate whether moderators could amplify or weaken the influences of hypothesized antecedents on knowledge hiding.

## 3 The nomological framework of knowledge hiding

### 3.1 Antecedents of knowledge hiding

#### 3.1.1 Benefits and knowledge hiding

Perceived justice can be summarized as people’s subjective evaluations of organizational fairness and equity in the resource allocation process (Gelens et al., 2013). Drawing on SET, high-quality exchange relationships promote one’s engagement in positive reciprocity rather than negative reciprocity, representing that individuals have an obligation to reciprocate others who give assistance to them in the process of social exchange (Kim et al., 2019). Jiang et al. (2017) suggested that employees’ perceptions of organizational fairness greatly promote their motivation to obey organizational norms and enhance their trust in organizations. Thus, they are more likely to benefit from organizational justice in social transactions and display a resistance toward knowledge-hiding behaviors based on the norms of reciprocity.

Leader member exchange (LMX) is highly rooted in the norms of reciprocity; thus, the performance of people with high-quality LMX is enhanced when effective supervisor support is made available through effective social exchange processes (Wayne et al., 1997). LMX also stimulates employees’ willingness to “payback” their supervisors’ advantageous treatments by avoiding deviant behaviors, such as knowledge hiding, to achieve their shared goals (He et al., 2022). Similarly, Seers et al. (1995) indicated that team member exchange (TMX) shares the same core value with LMX, that is, people are obligated to reciprocate favorable treatments and high-quality exchange relationships that are executed on behalf of fellow employees. Extant literature has explored how TMX influences knowledge hiding. For example, Tan et al. (2022) suggested that individuals engaged in effective social exchange relationships with co-workers are more responsive to their knowledge requests and exhibit less knowledge-hiding intentions.

Perceived social support (PSS) can be regarded as a cognitive appraisal of feeling connected and supported by others (Lakey and Cassady, 1990), which plays a crucial role in shaping one’s attitudinal and behavioral responses to different job-related situations. For example, Loi et al. (2014) argued that both perceived organizational support and co-worker support significantly contribute to stimulating promotive workplace behaviors. Such arguments can be well rationalized based on the norms of reciprocity. The perception of social support makes people feel obligated to return the advantageous treatments received from others, which extensively strengthens the interpersonal relationships in the workplace (Eisenberger et al., 2001). Therefore, perceived social support could prevent people from engaging in knowledge-hiding behaviors by cultivating good interpersonal climates and tight social bonds.

*Hypothesis 1*: Perceived justice, LMX, TMX, and PSS are negatively related to knowledge hiding.

#### 3.1.2 Costs and knowledge hiding

Tepper (2000, p.178) defined abusive supervision as “subordinates’ perceptions of the extent to which supervisors engage in the sustained display of hostile verbal and nonverbal behaviors, excluding physical contact.” Extant literature confirmed that abusive supervisors would enable their subordinates to suffer from unpleasant working experiences and lead to unfavorable behavioral responses (Fischer et al., 2021). Accordingly, Aryee et al. (2007) suggested that abusive supervision destroys the quality of social exchanges within organizations, especially between the leader and victim. The continuous psychological and mental harassment from abusive supervision stimulates one’s negative reciprocity beliefs in the workplace because they are forced to experience an annoying social cost (Lian et al., 2014). To respond to such destructive leadership, employees would restore their balance of exchanges by retaliating against the abusive supervisors and violating organizational norms, such as engaging in workplace deviant behaviors (Lian et al., 2014; Mitchell and Ambrose, 2007). Thus, abused subordinates are inclined to intentionally conceal their valuable expertise and skills from their supervisors to avoid further exploitation.

Relationship conflict is defined as the subjective evaluation of disagreements and incompatibilities among individuals regarding personal values and social issues beyond work tasks (Jehn et al., 1997), such as personality differences and interpersonal tensions. Relationship conflict generally has undesirable effects on personal affectivity and behavior (Choi et al., 2024; Peng et al., 2021), which enhances individuals’ willingness to benefit themselves over others. Drawing on SET, social exchanges between two interdependent parties require them to obey transaction rules that return the favor or harm to others in the same way as it was received (Kundi et al., 2023; Mitchell et al., 2012). Thus, the relationship conflict in dyads would make people generate a feeling that others, such as supervisors or co-workers, should be accountable for their potential losses in the process of exchanges. Moreover, Boz Semerci (2019) also justified the influence of relationship conflict on knowledge hiding through the lens of social exchange, as people in relationship conflict focus more on their personal interests by hiding knowledge from others in the workplace.

Pearson et al. (2000, p. 125) defined workplace incivility as “mistreatment that may lead to disconnection, breach of relationships, and erosion of empathy.” Workplace incivility adversely influences individuals’ work patterns, effectiveness, and ability to perform daily tasks (Pearson et al., 2000). Therefore, the return on injuries, such as engaging in counterproductive knowledge-related behaviors, is more preferred by individuals under the influence of workplace incivility. This is because retaliation is a more appropriate response to workplace mistreatments (Gouldner, 1960; Wu et al., 2014). For example, Anand et al. (2023) empirically identified that workplace incivility triggers one’s knowledge-hiding behaviors because of the violation of reciprocity norms, thus returning harm to harm. Similarly, workplace ostracism interferes with individuals’ perceptions of organizational surroundings, which, in turn, exerts a negative influence on work-related attitudinal and behavioral outcomes (Wu et al., 2012; Yan et al., 2014). Based on the norm of reciprocity, poor-quality interpersonal relations weaken people’s expectations of reciprocity in the social exchange process (Gouldner, 1960). To balance social costs with resource loss from workplace exclusion, ostracized individuals are more likely to exhibit knowledge-hiding behaviors.

Perceived competition refers to people’s perception that they need to outperform others within their organizations in terms of rewards, recognition, and status, along with a sense of hostility (Chaker et al., 2021). Continuous pressure from competition causes people to generate greater workplace tension and view colleagues as primary rivals. When people perceive competition around them, they are easily displaying withholding practices toward valuable resources to ensure their competitiveness (Dedman and Lennox, 2009). From the social exchange perspective, individuals expect to be well rewarded equivalently from the social exchange when they incur certain amount costs (Chen et al., 2009). Thus, people would exhibit a tendency to break reciprocal relationships and hold on to their knowledge and skills when they feel threatened by adverse competition (Oubrich et al., 2021).

Psychological entitlement can be summarized as a stable feeling that one deserves more or is entitled to more privileges than one’s peers (Campbell et al., 2004). When the requirements of inflated self-importance are not satisfied with the proclaimed rewards, psychologically entitled individuals generally assert that they are treated unfairly, as they do not get what they are seeking (Harvey and Harris, 2010). In this case, entitled people are more likely to violate conventional social norms and engage in vengeful behaviors toward the so-called “inequity” at work. For example, Khalid et al. (2020) empirically identified that entitled employees believe that organizations violate the norms of reciprocity and mistreat them in social transactions. The hatred on organizations and fellow employees would stimulate knowledge-hiding behaviors as retaliation.

*Hypothesis 2*: Abusive supervision, relationship conflict, workplace incivility, workplace ostracism, perceived competition, and psychological entitlement are positively related to knowledge hiding.

### 3.2 Outcomes of knowledge hiding

#### 3.2.1 Knowledge hiding and positive reciprocity outcomes

Gurteen (1998) stated that creativity and innovation represent the procedures of creating and implementing knowledge, through which the commercial value of knowledge is realized instead of it being confined to a laboratory. In the knowledge economy era, the speed of knowledge updates and the effectiveness of knowledge flows are important motivators for promoting creative and innovative performance (CIP) (Park et al., 2014; Wang and Wang, 2012). However, the existence of knowledge hiding within organizations destroys one’s intrinsic motivation to pursue further creativity and innovation because of triggering a reciprocal distrust loop (Černe et al., 2014). Therefore, knowledge hiding prevents people from obtaining the expertise required to create new ideas, because it damages social exchange relationships and hinders the information flows.

Task performance is another important outcome of knowledge hiding concerning job-related obligations and responsibilities (Singh, 2019). In accordance with SET, employees would like to shape their social relationships based on their own experiences acquired from workplace exchanges (Blau, 2017). The poor experiences from social transactions motivate people to breach the norms of reciprocity by acting against perceived unfavorable treatments, thus keeping a balance between giving and taking in functional systems (Chen et al., 2009). From the empirical perspective, Singh (2019) demonstrated that knowledge seekers adversely respond to knowledge hiders by exhibiting a non-cooperative attitude as a retaliation for their territoriality of knowledge. Besides, Moin et al. (2024) confirmed this finding as well, indicating that knowledge hiding hinders individuals’ capability to perform well on job-related affairs due to knowledge seekers’ reluctancy to return good for evil.

Extra-role behavior (ERB) refers to discretionary activities that go beyond job descriptions but contribute to enhancing organizational effectiveness (Leung, 2008), such as employees’ voice. Extra-role behavior is considerably suppressed because the prevalence of knowledge hiding fosters the trust crisis of exchange rules within organizations (Hameed et al., 2023). Intentionally concealing knowledge has been viewed as a form of anti-social behavior (Connelly and Zweig, 2015) and individuals are compelled to respond to knowledge hiding with lower prosocial motivation to protect their personal interests. From the social exchange perspective, individuals would like to reciprocate negative treatments with something of same value, thus, people are inclined to disengage from ERB until a balanced knowledge exchange is achieved (Cropanzano et al., 2017).

*Hypothesis 3: Knowledge hiding is negatively related to CIP, task performance, and ERB.*

#### 3.2.2 Knowledge hiding and negative reciprocity outcomes

Turnover intention refers to employees’ desire to leave their current job and look forward to finding a better one (Ahmad Saufi et al., 2023). Drawing on SET, the maintenance of a workplace relationship depends on interdependent parties involved in the social exchange process perceiving the relationship as beneficial and favorable (Blau, 2017; Cropanzano et al., 2017). In other words, employees generally behave in a similar manner to the way they are being treated within organizations. As instructed by Serenko and Bontis (2016), knowledge hiding generally acts as a motivator of turnover intention, resulting in significant tangible and intangible costs because of the loss of relational and human capital Thus, employees’ knowledge requests are not fulfilled, and they simultaneously experience a sense of exclusion, which increases the possibility of their departure from the organization.

Finally, this study investigates how knowledge hiding influences workplace deviance. Workplace deviance can be summarized as a subjective violation of organizational norms, thereby damaging people’s overall well-being and lowering organizational performance (Robinson and Bennett, 1995). As instructed by Ahmad et al. (2023), individuals prefer to maximize the benefits with the least cost in the process of social exchanges. If potential risks outweigh promised rewards, people easily exhibit deviant behaviors to avoid further loss. Several empirical studies (Arain et al., 2022; Singh, 2019) have identified that knowledge hiding adversely destroys mutual trust and reciprocal relationships among colleagues and individuals, thus exhibiting higher levels of workplace deviance.

*Hypothesis 4*: Knowledge hiding is positively related to turnover intentions and workplace deviance.

### 3.3 Moderators

The first moderators were based on the cultural environment in which the primary studies were conducted. As noted by Xiong et al. (2021), cultural variations may influence the ideas and practices of knowledge-related behaviors. For example, collectivism encourages individuals to obey organizational obligations, work for shared goals, and even make personal sacrifices for the collective benefits (House et al., 2004); whereas individualists generally behave in accordance with their personal values and attitudes rather than widely accepted social norms, along with an emphasis on personal interests (Xiong et al., 2021). In the case of knowledge hiding, personal values developed from social culture tend to greatly regulate individuals’ communication styles and work behaviors, especially how they deal with knowledge hiding (Boz Semerci, 2019). Thus, Benefit, as well as Cost, may exert significantly differentiated effects on knowledge hiding when comparing collectivism and individualism. Therefore, we explored the following question:

*Research question 1: Does collectivism culture moderate the relationships between Benefit and knowledge hiding relationships and Cost and knowledge hiding?*

Power distance is defined as the extent to which people anticipate and concur with the unequal distribution of power within an organization or society (Hofstede, 2001). In a higher power distance culture, individuals are more likely to advocate an internal hierarchy and hold on to the belief that they should not challenge or question the decisions of those in charge (De Luque and Sommer, 2000; Tyler et al., 2000). By contrast, Shao et al. (2013) indicated that, within a lower power distance culture, there is a notable inclination toward power equality and procedural justice when it comes to any existing unfair treatment. Therefore, we assumed that people’s attitudes toward knowledge hiding would not only be shaped by external environments, but also by their power cognition, such as tolerance of power inequality. As such, the effects of Benefit and Cost on knowledge hiding may vary across various levels of power distance culture. Therefore, we explored the following question.

*Research question 2: Does power distance culture moderate the relationships between Benefit and knowledge hiding and Cost and knowledge hiding?*

In the knowledge economy era, knowledge hiding has been examined differently across industries, and industry differences influence the way in which it develops and functions. Accordingly, this meta-analysis also examined the moderating role of knowledge intensity in the industries from which data were collected. From a knowledge-based perspective, knowledge intensity can be explained as the extent to which an organization relies on knowledge to develop competitive advantages, maintain survival, and achieve commercial value (Andreeva and Kianto, 2011). Knowledge has also been found to have a positive relationship with the knowledge creation and sharing processes, especially in knowledge-intensive industries (Andreeva and Kianto, 2011). Therefore, we assumed that knowledge-intensive organizations would behave differently when they notice the effects of Benefit and Cost on knowledge hiding compared with less knowledge-intensive organizations. Then, we examined the following question:

*Research question 3: Does knowledge intensity moderate the relationships between Benefit and knowledge hiding and Cost and knowledge hiding?*

In extant literature, different scales have been adopted to measure knowledge hiding. For example, Peng (2012), Serenko and Bontis (2016), and Rhee and Choi (2017) developed their own scales to evaluate the magnitude of the knowledge-hiding phenomenon, with an emphasis on its deceptive nature. Further, Connelly et al. (2012) provided the most typical measurement tool for knowledge hiding by first introducing three dimensions: evasive hiding, playing dumb, and rationalized hiding, which exhibited a more holistic depiction of knowledge hiding. Unlike other scales, Connelly et al.’s (2012) scale justifies the non-deceptive nature of knowledge hiding, indicating that it is not always negative and counterproductive (Offergelt et al., 2019). Given the distinctiveness of knowledge-hiding scales, we assume that the variations across studies may originate from the adoption of different scales to measure knowledge hiding. Hence, in this study, the moderating role of knowledge-hiding measures was examined and put forward the following research question was proposed:

*Research question 4: Does the way in which knowledge hiding is measured moderate the relationships between Benefit and knowledge hiding and Cost and knowledge hiding?*

## 4 Research methodology

### 4.1 Literature search and inclusion criteria

**Figure 2:**
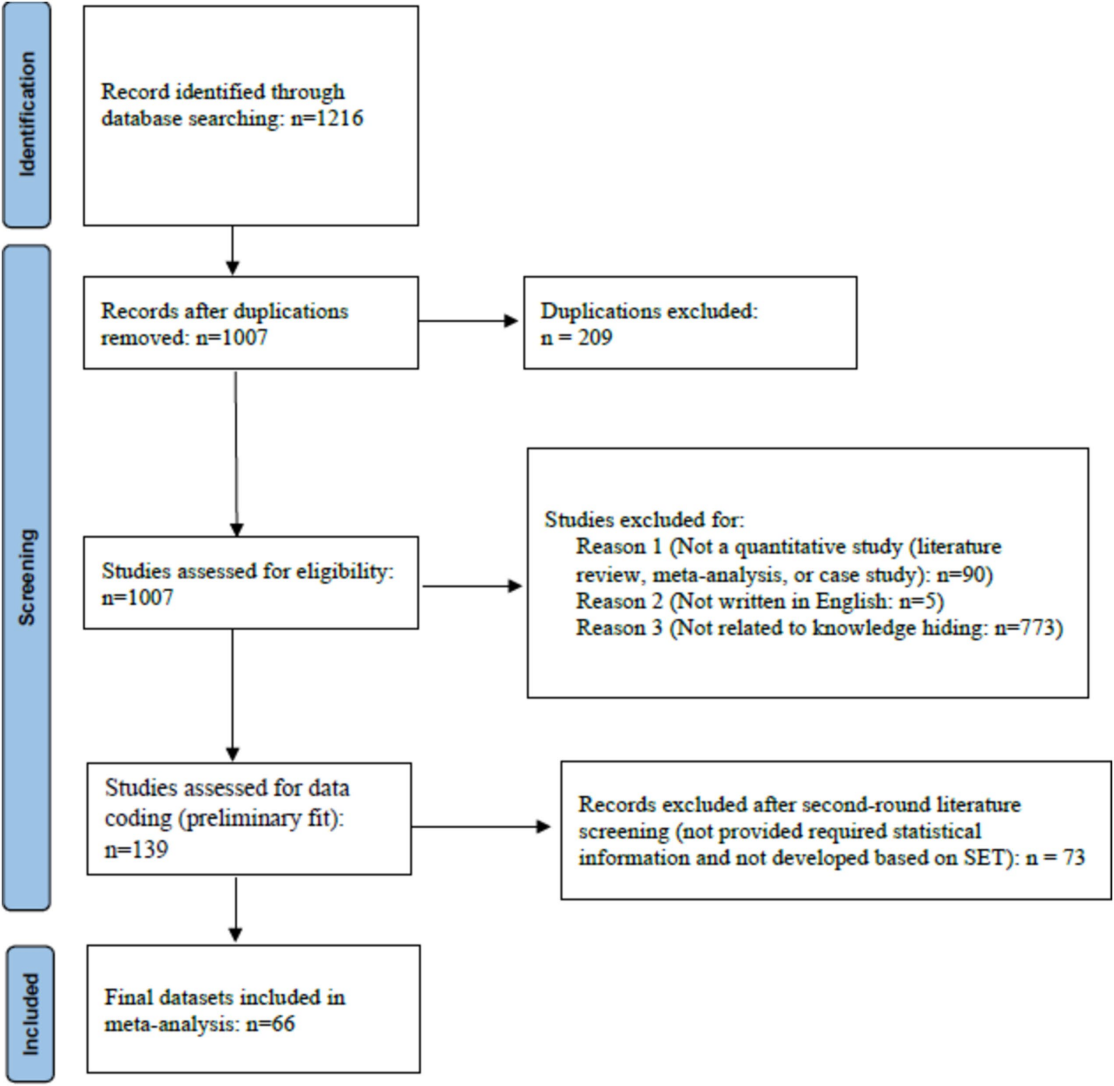
Flowchart of literature searching and screening.

To identify relevant studies for the meta-analysis, we conducted a thorough search on the correlates of knowledge hiding from seven online academic sources: Google Scholar, Web of Science, ProQuest, ScienceDirect, Elsevier, Sage, and Taylor & Francis Online. Search items included a combination of “knowledge hiding,” “hide knowledge,” “knowledge hoarding,” “knowledge withholding,” “withhold knowledge,” “social exchange theory,” and “norm of reciprocity.” Moreover, we also consulted the reference lists of previous systematic literature reviews and meta-analyses in the field of knowledge hiding. Finally, this online search was supplemented by an additional examination of theses and dissertations, to include all relevant literature to the maximum extent possible. Figure 2 shows a flowchart of the literature searching and screening processes.

For inclusion in the meta-analysis, each primary study should meet five established criteria: (1) Cronbach’s alpha and at least one bivariate correlation between knowledge hiding and its correlates were provided; (2) the study was written in English; (3) field samples of employed respondents were used; (4) if a study included two independent samples, the data from these two samples were coded independently; (5) meta-analytic analyses were conducted only for pairwise relationships that were examined by at least two empirical studies. Therefore, our preliminary search identified 1,216 primary studies that matched the keywords from seven online sources. Of these, 139 studies were selected after excluding papers that were not relevant, not in English, not quantitative research, or used in experimental samples. In the second round of the literature screening, we excluded studies that did not provide the required statistical information or were not developed based on SET. Finally, 66 primary studies (60 journal articles and 6 theses/dissertations) from 2014 to 2024, comprising 20,603 participants, were included in the current meta-analysis.

Two of the authors independently coded each primary study included in the meta-analysis. The coding procedure was initially tested with a sample of 10 studies and their preliminary results were shared to ensure accuracy. The two authors carefully double-checked the statistical values from primary studies, such as inter-correlations, number of respondents, and reliability coefficients. The initial inter-rater agreement of data coding was 91%. If the two authors could not reach an agreement, a third author was asked to help them decide. Finally, all discrepancies were resolved, and a 100% consensus was reached.

### 4.2 Meta-analytic model

Schmidt and Hunter’s (2015) random-effects model was introduced to correct sampling and measurement errors for all observed correlations by incorporating two key values: sample size and Cronbach’s alpha. The random-effects model allows population parameters to vary across studies owing to variable respondents, interventions, and other types of unexplained heterogeneity (Borenstein et al., 2010; Higgins et al., 2009). In this meta-analysis, we reported the number of primary studies (K) that included the relationships between knowledge hiding and its correlates, and the corresponding total sample size (N). Additionally, we reported sample-size weighted mean uncorrected correlation ($\bar{r}$) and estimated true score correlation corrected for measurement and sampling errors (ρ), as well as their respective standard deviations ($\bar{SD}$ and $SD_{\rho }$). Moreover, 95% confidence intervals and 80% credibility intervals were computed to assess the statistical significance of true-score correlations and the potential moderation effects in the meta-analysis (Miao et al., 2016). Finally, we calculated the percentage of observed variance attributed to statistical artifacts (Var%), such as sampling and measurement errors (Schmidt and Hunter, 2015).

This study also examined the potential mediating effects of knowledge hiding in the relationships between its antecedents and outcomes through MASEM, which incorporates the advantages of meta-analytic techniques and the structural equation modeling (SEM) framework. The MASEM technique is a two-stage quantitative research method that summarizes pooled effect sizes and their standard deviations from this current meta-analysis and previous relevant meta-analytic studies to develop average correlation matrices that can be further adopted to fit the path analysis in SEM to detect the validity of the proposed hypotheses (Cheung, 2014; Cheung and Cheung, 2016). The MASEM can effectively boost sample sizes by accumulating different samples, which enhances the interpretive power of the structural model and the precise assessment of estimates, rather than either technique alone.

Finally, to rationalize observed heterogeneity in the current meta-analysis, we conducted a moderation analysis to assess whether national culture, organizational context, and knowledge-hiding measures could foster or attenuate the influence of the proposed predictors on knowledge hiding. Following the research protocol of Nguyen et al. (2019), we only examined the moderating effects when a moderator was accompanied by at least two primary studies on each side. In the field of knowledge management, collectivism and power distance are two cultural indices used to evaluate how national cultures affect individuals’ social cognition and behaviors (Boz Semerci, 2019; Chiu et al., 2018). In terms of the cultural context, we coded the national information of each primary study and assigned values of collectivism and power distance to each country based on the global scores provided by House et al. (2004). We then used the mean value to code these countries into higher or lower subgroups of collectivism and power distance. Similarly, we conducted a thorough investigation of the included studies to obtain pertinent details regarding knowledge intensity and knowledge-hiding measures. If there was a lack of information regarding the role of moderators, the primary study was coded as “NA” and subsequently excluded from the moderation analysis.

## 5 Results

### 5.1 Publication bias

Publication bias is defined as “an editorial predilection for publishing particular findings, e.g., positive results, which leads to the failure of authors to submit negative findings for publication” (Thornton and Lee, 2000, p. 207). Some statistical methods were also adopted to identify whether publication bias could disturb the synthesized results as follows. Egger’s value was chosen to examine the existence of publication bias (Egger et al., 1997); all *p*-values for Egger’s tests were greater than 0.05, indicating no true publication bias for pairwise relationships between knowledge hiding and its correlates.

### 5.2 Heterogeneity test

At the beginning of a meta-analysis, it is necessary to conduct a test of heterogeneity, which reflects the extent to which heterogeneity may influence the overall conclusions from the collection of primary studies (Higgins and Thompson, 2002). Two typical methods were used to examine true heterogeneity in the current meta-analysis. The *Q*-test was first introduced in 1954. If the *p*-value of the *Q*-test was significant at the 95% confidence level, we rejected the homogeneous null hypothesis that all selected studies are identical, indicating that there is a true heterogeneity across the included studies (Lin, 2020). However, the *Q* value is highly influenced by the number of included studies, as it exhibits poor statistical power to identify the existence of heterogeneity with a small number of primary studies (Huedo-Medina et al., 2006). Therefore, we cannot rely on only one specific method to assess true heterogeneity in the meta-analysis. I-squared is also used to compensate for the shortcomings of the Q-test, which is not sensitive to the number of included studies. Table 1 shows the results of the heterogeneity test for each relationship in the meta-analysis. Most Q-values for pairwise relationships, except for TMX, were significant at the 95% confidence level (*p* < 0.05), indicating the presence of true heterogeneity between studies. The same results were found using I-squared. In conclusion, because of the presence of true heterogeneity in the hypothesized relationships, the random-effects model was the most appropriate statistical technique for current meta-analysis. It exhibits a higher acceptance of between-study variance rather than fixed-effects model, assuming that “the true population effect size could vary from study to study” (Montazemi and Qahri-Saremi, 2015, p. 218).

**Table 1:** Heterogeneity test.

Pairwise relationship	*K*	Q-statistics	*p*-value	$I^{2}$
Abusive supervision > KH	8	117.058	0.000	94.020
Perceived competition > KH	3	80.962	0.000	97.530
Relationship conflict > KH	8	70.256	0.000	90.036
Perceived justice > KH	6	64.603	0.000	92.260
Workplace incivility > KH	7	77.115	0.000	92.219
Workplace ostracism > KH	4	28.544	0.000	89.490
LMX > KH	5	41.703	0.000	90.408
TMX > KH	2	0.004	0.951	0
PSS > KH	9	29.766	0.000	73.124
Psychological entitlement > KH	3	135.036	0.000	98.519
KH > CIP	12	137.030	0.000	91.973
KH > Workplace deviance	6	112.043	0.000	95.537
KH > task performance	6	52.788	0.000	90.528
KH > turnover intentions	5	142.876	0.000	97.200
KH > ERB	4	13.253	0.006	77.364

K: independent samples; LMX: leader-member exchange; TMX: team-member exchange, PSS: perceived social support; CIP: creative and innovative performance; ERB: extra-role behavior; Q-statistics and $I^{2}$: statistical indices for heterogeneity.

### 5.3 Main effects between knowledge hiding and its correlates

Table 2 shows the overall meta-analytic results for the hypothesized relationships between knowledge hiding and its correlates (10 relationships between knowledge hiding and its antecedents and 5 relationships between knowledge hiding and its outcomes). Cohen (1992) proposed specific values for Pearson’s R, which serve as thresholds for determining the magnitude of the effect size. To classify the effects as small, medium, and large, Cohen (1992) suggested using R-values of 0.10, 0.30, and 0.50. From the perspective of antecedents, workplace incivility (*ρ* = 0.572, *k* = 7), is strongly and positively associated with knowledge hiding. Abusive supervision (ρ=0.453, *k* = 8), relationship conflict (ρ = 0.405, *k* = 8), and workplace ostracism (ρ = 0.417, *k* = 4) are moderately and positively associated with overall knowledge hiding. Conversely, TMX (ρ = −0.590, *k* = 2) is strongly and negatively related to knowledge hiding, while LMX (ρ = −0.310, *k* = 8) and perceived justice (ρ = −0.364, *k* = 6) are moderately and negatively associated with knowledge hiding. PSS (ρ = −0.269, *k* = 9) is weakly and negatively related to knowledge hiding. However, perceived competition and psychological entitlement do not show significant correlations with knowledge hiding. Thus, we can conclude that Hypothesis 1 is fully supported, and Hypothesis 2 is partially supported.

**Table 2:** Meta-analytic results of antecedents and consequences of overall knowledge hiding.

Antecedents of KH	*N*	*K*	$\bar{r}$	$\bar{SD}$	ρ	$SD_{\rho }$	$CI_{L}$	$CI_{U}$	$CV_{L}$	$CV_{U}$	%Var
Abusive supervision	2,384	8	0.409	0.183	0.453	0.215	0.267	0.638	0.148	0.757	5.980
Perceived competition	968	3	0.410	0.311	0.502	0.351	−0.380	1.380	−0.159	1.160	2.470
Relationship conflict	2,637	8	0.367	0.143	0.405	0.160	0.264	0.546	0.179	0.631	9.964
Perceived justice	1,316	6	−0.319	0.217	−0.364	0.240	−0.626	−0.102	−0.718	−0.010	7.740
Workplace incivility	2,085	7	0.511	0.148	0.572	0.166	0.412	0.732	0.333	0.812	7.781
Workplace ostracism	1,056	4	0.35	0.139	0.417	0.193	0.092	0.742	0.100	0.733	10.510
LMX	3,129	8	−0.262	0.155	−0.310	0.162	−0.453	−0.167	−0.540	−0.080	9.592
TMX	830	2	−0.535	0.023	−0.590	0.00	−0.612	−0.569	−0.59	−0.59	26008.702
PSS	1,825	9	−0.233	0.136	−0.269	0.125	−0.381	−0.157	−0.443	−0.095	26.876
Psychological entitlement	1,152	3	0.187	0.400	0.202	0.451	−0.927	1.330	−0.648	1.050	1.481
**Outcomes of KH**											
CIP	4,413	12	−0.355	0.157	−0.398	0.174	−0.514	−0.283	−0.636	−0.161	8.027
Workplace deviance	2,026	6	0.447	0.191	0.493	0.225	0.251	0.735	0.160	0.826	4.463
Task performance	1,859	6	−0.420	0.144	−0.465	0.161	−0.643	−0.286	−0.703	−0.226	9.472
Turnover intention	1,616	5	0.056	0.332	0.061	0.367	−0.402	0.524	−0.502	0.624	2.800
ERB	1,126	4	−0.199	0.135	−0.235	0.121	−0.455	−0.016	−0.434	−0.037	22.636

K: number of primary studies included in the meta-analysis; N: total numbers of respondents; $\bar{r}$: sample-size weighted mean uncorrected correlation; $\bar{SD}$: standard deviation of sample-size weighted mean uncorrected correlation; ρ: estimated true score correlation corrected for measurement and sampling errors; $SD_{\rho }$: standard deviation of estimated true score correlation corrected for measurement and sampling errors; CL: confidence intervals; CV: credibility intervals; Var%: percentage of observed variance attributed to statistical artifacts; LMX: leader-member exchange; TMX: team-member exchange; PSS: perceived social support; CIP: creative and innovative performance; ERB: extra-role behavior.

From the perspective of outcomes, knowledge hiding is negatively associated with CIP (ρ = −0.398, *k* = 12), task performance (ρ = −0.465, *k* = 6), and ERB (ρ = −0.235, *k* = 4), indicating that Hypothesis 3 is fully supported. Among these three negative relationships, knowledge hiding is moderately related to CIP and task performance, but knowledge hiding is weakly associated with ERB. By contrast, knowledge hiding is moderately and positively related to workplace deviance (ρ = 0.493, *k* = 6). Finally, there is no bivariate relationship between knowledge hiding and turnover intentions, indicating that the Hypothesis 4 is partially supported.

### 5.4 Mediation analysis

In addition to investigating knowledge hiding as an antecedent or outcome of a specific factor that we hypothesized, this meta-analysis also examines the intermediatory role of knowledge hiding using MASEM techniques. We adopted three criteria to choose appropriate antecedents and outcomes to be included in the mediation analysis: (1) the constructs should have a significant relationship with knowledge hiding; (2) to construct correlation matrices as the data input for mediation analysis, pooled effect sizes (ρ) should be found in previous meta-analyses or can be calculated from independent primary studies; and (3) an antecedent or outcome needs to be examined in at least four studies (Škerlavaj et al., 2023). Accordingly, we included four antecedents (abusive supervision, perceived justice, workplace incivility, PSS, and LMX) and four outcomes (CIP, task performance, workplace deviance, and ERB) in the mediation analysis.

Before running the MASEM, we assessed the goodness of fit between the measurement model and alternative models. As the relationships between knowledge hiding and its correlates were developed based on the SET, we simultaneously included four antecedents and one outcome in the mediation analysis. To examine the mediation effect, we compared a partial mediation model with a total effect model and full mediation model (Miao et al., 2016). When CIP was regarded as the criterion variable in the measurement model, the total effect model (χ^2^ = 1027.483, df = 6, *p* = 0.000, root mean square error of approximation (RMSEA) = 0.300, comparative fit index (CFI) = 0.494, Tucker-Lewis index (TLI) = 0.072, standardized root mean square residual (SRMR) = 0.275) and full mediation model (χ^2^ = 818.442, df = 5, p = 0.000, RMSEA = 0.294, CFI = 0.597, TLI = 0.113, SRMR = 0.156) did not show a good model fit compared with the partial mediation model (χ^2^ = 0.000, CFI = 1.000, TLI = 1.000, SRMR = 0.000). When task performance, workplace deviance, and ERB were included in the mediation analysis with the four antecedents remaining constant, similar results were found for the partial mediation model (χ^2^ = 0.000, CFI = 1.000, TLI = 1.000, SRMR = 0.000). Thus, we chose the partial mediation model for the mediation analysis.

**Figure 3:**
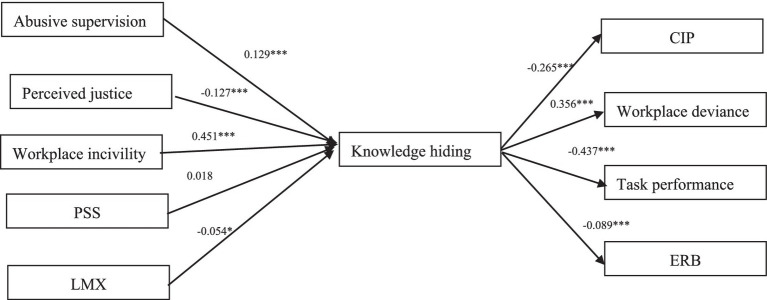
Mediation model.

Table 3 and Figure 3 present the results of the mediation analyses. Knowledge hiding can act as a mediator in most indirect relationships. Specifically, it plays a mediating role in the relationship between abusive supervision on the one hand, and CIP (β= − 0.034, [−0.049, −0.021]), task performance (β= − 0.056, [−0.078, −0.036]), ERB (β= − 0.012, [−0.020, −0.005]), and workplace deviance (β=0.046, [0.029, 0.064]) on the other. Similarly, the direct effects of workplace incivility on CIP (β= − 0.120), task performance (β= −0.197), ERB (β= − 0.040), and workplace deviance (β=0.160) are also mediated by knowledge hiding. The indirect estimates of knowledge hiding in the relationship between perceived justice, LMX, and the proposed outcomes (CIP, task performance, ERB and workplace deviance) are all significant. However, the underlying mechanisms between PSS and CIP, task performance, ERB, and workplace deviance could not be elucidated through knowledge hiding.

**Table 3:** Mediation analysis.

Path	Estimate	SE	$CL_{L}$	$CL_{U}$
AS-KH-CIP	−0.034	0.007	−0.049	−0.021
AS-KH-Task performance	−0.056	0.011	−0.078	−0.036
AS-KH-ERB	−0.012	0.004	−0.020	−0.005
AS-KH-Workplace deviance	0.046	0.009	0.029	0.064
Perceived justice-KH-CIP	0.034	0.007	0.021	0.048
Perceived justice-KH-task performance	0.055	0.010	0.036	0.076
Perceived justice-KH-ERB	0.011	0.004	0.005	0.019
Perceived justice-KH-workplace deviance	−0.045	0.009	−0.063	−0.029
Incivility-KH-CIP	−0.120	0.011	−0.142	−0.098
Incivility-KH-task performance	−0.197	0.014	−0.225	−0.171
Incivility-KH-ERB	−0.040	0.011	−0.062	−0.019
Incivility-KH-workplace deviance	0.160	0.013	0.136	0.186
PSS-KH-CIP	−0.005	0.006	−0.017	0.007
PSS-KH-Task performance	−0.008	0.010	−0.027	0.011
PSS-KH-ERB	−0.002	0.002	−0.006	0.002
PSS-KH-workplace deviance	0.006	0.008	−0.009	0.022
LMX-KH-CIP	0.014	0.006	0.002	0.058
LMX-KH-task performance	0.024	0.010	0.004	0.044
LMX-KH-ERB	0.005	0.002	0.001	0.010
LMX-KH-workplace deviance	−0.019	0.008	−0.036	−0.003

AS: abusive supervision; KH: knowledge hiding; CIP: creative and innovative performance; ERB: extra-role behavior; PSS: perceived social support; LMX: leader-member exchange; SE: standard error; CL: confidence intervals.

### 5.5 Moderation analysis

To examine the source of heterogeneity, a subgroup analysis was adopted to investigate how potential moderators influenced knowledge hiding. As instructed by Nguyen et al. (2019), we calculated the between-group variance (Q-statistics) to identify whether moderators could make significant differences in specific pairwise relationships.

Regarding national culture, as shown in Table 4, we found that the effect size of the abusive supervision-knowledge hiding relationship was larger in low collectivist cultures (*ρ* = 0.588) than in high ones (*ρ* = 0.310). In addition, this study also identified the moderating role of power distance on the relationship between abusive supervision and knowledge hiding at the 90% confidence level, in which the effect size was larger in lower power distance cultures (*ρ* = 0.505) than in their higher counterparts (*ρ* = 0.275). Similarly, power distance also moderated the relationship between LMX and knowledge hiding at the 95% confidence level. The inhibiting effect of LMX on knowledge hiding was greater in lower power distance cultures (*ρ* = −0.454) than in higher power distance cultures (*ρ* = −0.282).

**Table 4:** Subgroup analysis.

Variable	*k*	*N*	*ρ*	$CI_{L}$	$CI_{U}$	*Z*-value	Q-statistics	*p*-value
**Antecedents**								
**Abusive supervision**								
Lower collectivism	3	829	0.588	0.511	0.655	12.001***	13.780	0.000***
Higher collectivism	5	1,555	0.310	0.168	0.439	4.155***		
Lower power distance	5	1,382	0.505	0.363	0.624	6.217***	3.589	0.058^+^
Higher power distance	3	1,002	0.275	0.059	0.466	2.477*		
Lower knowledge intensity	1	241	NA	NA	NA	NA	/	/
Higher knowledge intensity	4	1,141	0.499	0.315	0.647	4.840***		
Connelly measures	5	1,573	0.460	0.350	0.558	7.358***	0.323	0.570
Other measures	3	811	0.361	−0.009	0.645	1.913^+^		
**Relationship conflict**								
Lower collectivism	4	1,322	0.425	0.307	0.530	6.519***	0.593	0.441
High collectivism	4	1,315	0.345	0.164	0.504	3.636***		
Lower power distance	3	788	0.385	0.152	0.577	3.145**	0.000	0.991
Higher power distance	5	1,849	0.386	0.262	0.497	5.762***		
Lower knowledge intensity	3	1,231	0.297	0.196	0.391	5.595***	15.026	0.000***
Higher knowledge intensity	4	1,168	0.498	0.454	0.540	18.599***		
Connelly measure	6	2,019	0.342	0.227	0.447	5.582***	7.156	0.007**
Other measures	2	618	0.507	0.446	0.563	13.816***		
**Workplace incivility**								
Lower collectivism	2	670	0.530	0.229	0.738	3.245**	0.329	0.566
Higher collectivism	3	861	0.466	0.344	0.538	7.744***		
Lower power distance	2	753	0.405	0.334	0.471	10.246***	1.774	0.183
Higher power distance	3	778	0.542	0.344	0.693	4.804***		
Lower knowledge intensity	2	694	0.574	0.289	0.766	3.590***	0.195	0.659
Higher knowledge intensity	3	1,078	0.503	0.276	0.677	4.021***		
Connelly measures	6	1,862	0.526	0.400	0.632	7.119***	/	/
Other measures	1	223	NA	NA	NA	NA		
**LMX**								
Lower collectivism	1	323	NA	NA	NA	NA	/	/
Higher collectivism	5	2,290	−0.325	−0.468	−0.166	−3.894***		
Lower power distance	2	653	−0.454	−0.513	−0.390	−12.447***	4.195	0.041*
Higher power distance	4	1,960	−0.282	−0.434	−0.116	−3.267**		
Lower knowledge intensity	2	546	−0.354	−0.496	−0.193	−4.157***	1.239	0.266
Higher knowledge intensity	4	2,149	−0.227	−0.382	−0.061	−2.661**		
Connelly measures	4	1,062	−0.240	−0.392	−0.074	−2.814**	0.622	0.430
Other measures	4	2,067	−0.339	−0.509	−0.143	−3.317**		
**PSS**								
Lower collectivism	1	199	NA	NA	NA	NA	/	/
Higher collectivism	5	941	−0.283	−0.415	−0.139	−3.773***		
Lower power distance	3	596	−0.218	−0.362	−0.065	−2.767**	2.025	0.155
Higher power distance	3	544	−0.361	−0.480	−0.228	−5.080***		
Lower knowledge intensity	1	199	NA	NA	NA	NA	/	/
Higher knowledge intensity	4	851	−0.208	−0.340	−0.068	−2.895**		
Connelly measure	6	1,281	−0.190	−0.274	−0.103	−4.236***	4.530	0.033*
Other measures	3	544	−0.361	−0.480	−0.228	−5.080***		

K: number of primary studies included in the subgroup analysis; N: total numbers of respondents; ρ: sample-size weighted and reliability corrected population correlation; CL: confidence intervals; Q-statistics and *p*-values: significance of heterogeneity. ^+^*p* < 0.1.

*p < 0.05; **p < 0.01; ***p < 0.001.

From a knowledge-based perspective, the importance of industrial-level knowledge intensity has also been considered in knowledge management research because dependence on knowledge in productive activities is the main source of competitive advantage (Autio et al., 2000). As OECD (2006) suggested, knowledge-intensive industries include research and development institutions, information technology and communication services, legal services, financing, advertising, and market-related services, among others. Therefore, we assume that knowledge intensity moderates the relationships between knowledge hiding and its antecedents. In this case, the relationship between relationship conflict and knowledge hiding was moderated by knowledge intensity. Interpersonal conflict in more knowledge-intensive industries (*ρ* = 0.498) could have a greater stimulating influence on employees’ knowledge-hiding behaviors than in less knowledge-intensive industries (*ρ* = 0.297).

Finally, we investigated the moderating role of knowledge-hiding measures. As knowledge hiding has generally been measured using the scale developed by Connelly et al. (2012) in recent studies, it is worth exploring whether different measures of knowledge hiding could affect the research findings. The results indicated that most pairwise relationships between knowledge hiding and its correlates did not vary significantly across different knowledge-hiding measures, except for the relationships between relationship conflict and knowledge hiding, and between PSS and knowledge hiding. Among a few exceptions, the scale developed by Connelly et al. (2012) did not show greater statistical power in measuring knowledge hiding than other measures.

## 6 Discussion

First, this meta-analysis provides a quantitative summary of the relationships between knowledge hiding and its correlates from a social exchange perspective based on literature from 2014 to 2024. Ten antecedents were categorized into two subgroups: “Benefit” and “Cost,” in accordance with the norm of reciprocity, which is regarded as the main tenet of SET (Gouldner, 1960). Considering the antecedents of knowledge hiding, perceived justice, LMX, TMX, and PSS showed negative relationships with knowledge hiding, while abusive supervision, relationship conflict, workplace incivility, and workplace ostracism showed positive relationships with knowledge hiding. Among these antecedents, the magnitude of effect sizes for “Cost” is generally greater than that for “Benefit” in predicting knowledge hiding. This is consistent with the findings of a previous meta-analysis in the field of knowledge hiding (Arain et al., 2024), which indicated that negative events generally have a greater impact on individuals’ negative behavior than positive ones (Chiaburu et al., 2013). In addition, based on SET and the norm of reciprocity, the consequences of knowledge hiding were divided into positive and negative reciprocity outcomes. Similar results were identified: knowledge hiding exerted a stronger influence on negative reciprocity outcomes, such as workplace deviance, than on positive reciprocity outcomes, such as CIP, task performance, and ERB.

Second, this study examined the intermediary role of knowledge hiding, which highlighted the reciprocal loop of knowledge hiding from the social exchange perspective. As expected, the results indicated that knowledge hiding connected the relationships between abusive supervision, perceived justice, workplace incivility, LMX, and all selected outcomes (CIP, task performance, ERB and workplace deviance). Unfortunately, knowledge hiding could not mediate the relationship between PSS and the four selected outcomes. This can be explained by two reasons: (1) there might exist a methodological limitation in the mediation analysis, which collected pooled effect sizes from published meta-analyses and caused the loss of important information; (2) the indirect relationships between PSS, CIP, task performance, ERB, and workplace deviance via knowledge hiding might not be robust enough, as previous studies exhibited conflicting results on the relationship between PSS and knowledge hiding. For example, Batistič and Poell (2022) indicated that perceived organizational support (a form of PSS) was significantly related to knowledge hiding, whereas Alnaimi and Rjoub (2021) argued that there was no significant relationship between perceived organizational support and knowledge hiding.

Third, the moderating roles of collectivism and power distance were also demonstrated regarding the relationships between knowledge hiding and its correlates, suggesting that cultural differences also affect people’s knowledge-related behaviors by shaping their shared goals and power cognition. By investigating of collectivism and power distance, we found that the primary studies included in this meta-analysis were generally conducted in Asian countries, especially those characterized by higher levels of collectivism and power distance. This is consistent with the findings of Oliveira et al. (2021) regarding the regional distribution of knowledge-hiding research in recent years, with publications from non-eastern perspectives increasing slowly.

Finally, in addition to substantive moderators, this study also examined whether the research methodology makes a difference. Knowledge intensity and knowledge-hiding measures were explored as the methodological moderators. As for the research context, knowledge intensity moderated the relationship between relationship conflict and knowledge hiding, indicating that the relative importance of knowledge in different industries also influenced the severity of knowledge hiding. To examine the distinctiveness of Connelly et al.’s (2012) scale, we categorized studies on knowledge hiding into two subgroups: those using Connelly et al.’s (2012) scale and those using other scales. There were significant differences between the two subgroups in the relationships between relationship conflict, PSS, and knowledge hiding. However, the other measures exhibited stronger statistical power than the scale of Connelly et al. (2012). This result can be justified through the inclusion of rationalized hiding, which justifies the non-deceptive nature of knowledge hiding. The existence of positive aspects in knowledge hiding would make the values based on Connelly et al.’s (2012) scale less negative than those obtained from the other measures.

## 7 Theoretical contributions and practical implications

This meta-analysis contributes to the understanding of knowledge hiding in several ways. First, as an extension of the recent meta-analyses in the field of knowledge hiding (Arain et al., 2024; Škerlavaj et al., 2023), it established a new nomological framework of knowledge hiding, which successfully synthesized the reciprocal loop of this phenomenon in organizations from the social exchange perspective. By identifying knowledge hiding as a social exchange-based construct, the underlying mechanisms of knowledge hiding can be explored through individuals’ commitment to workplace fairness and adherence to reciprocal norms (O’Boyle et al., 2012; Xiao and Cooke, 2019). In terms of antecedents, dyadic relationships, such as horizontal interactions between co-workers and vertical interactions between supervisors and their subordinates, are critical to knowledge hiding. Its dynamics are also highly associated with the quality of personal social networks, and their position at both ends of the knowledge exchange spectrum also determines their attitudes toward knowledge hiding. Specifically, perceptions of reciprocal relationships contribute to the development of beneficial social exchanges that prevent people from engaging in knowledge-hiding behaviors, whereas perceptions of imbalanced social exchanges motivate people to violate social norms and prioritize personal interests, which increases the possibility of knowledge hiding.

Further, in terms of consequences, knowledge hiding due to failure in social exchanges weakens the internal solidity of relationships, groups, and organizations (Lawler, 2001), leading to negative reciprocation of undesirable behaviors in the workplace. In addition, the mediation analysis of knowledge hiding ensures the causalities between knowledge hiding and its antecedents and consequences, which provides a more comprehensive picture of the knowledge hiding loop between knowledge seekers and hiders. In conclusion, this study reveals the extent to which SET could truly depict and interpret the knowledge-hiding phenomenon.

In response to the appeal of Xiao and Cooke (2019), cultural-related constructs also matter significantly as boundary conditions in shaping people’s knowledge-hiding behaviors by adopting a cross-cultural perspective (Batistič and Poell, 2022). Concerning the moderating roles of cultural dimensions, our findings highlight the importance of integrating the knowledge-hiding literature with current cultural theories, such as Hofstede’s cultural framework (Xiao, 2024), which contributes to the establishment of important theoretical implications and effectively extends the generalizability of current meta-analytic findings on knowledge hiding across nations and cultures. Specifically, the collectivism and power distance in this meta-analysis could exert additional influences on knowledge hiding beyond the identified antecedents by shaping their cognitive appraisals of shared values and power distribution. Cultural-specific interpretations of knowledge hiding can help us explore how knowledge hiding is conceptualized and implemented in diverse cultures, and this investigation of cultural differences contributes to understanding the nuances of knowledge-hiding mechanisms among various social contexts. In addition, the moderating role of knowledge intensity suggests that knowledge hiding is a complex social phenomenon that fluctuates across industries, and knowledge concentration influences the way in which people react to knowledge hiding. Finally, this study indicates the unique characteristics and differential validity of Connelly et al.’s (2012) scale relative to others by highlighting the double-edged aspects of knowledge hiding. By identifying related yet different dimensions of knowledge hiding, a more comprehensive and practical measurement tool is introduced to knowledge-hiding research.

This study makes several practical contributions. Based on the nomological framework of knowledge hiding, the meta-analytic findings suggest that negative events have stronger power in fostering knowledge hiding than positive events. Therefore, top executives should be cautious about implementing managerial interventions to deal with destructive leadership and workplace mistreatment from supervisors and co-workers to inhibit the occurrence of knowledge hiding, especially in knowledge-intensive industries, which would contribute to mitigating the detrimental consequences on employees’ attitudes, behaviors, and job performance. In particular, leaders should be provided with leadership development programs to improve their abilities in drawing up effective knowledge management practices to address the knowledge-hiding phenomenon in organizations. For example, leaders should take time and make efforts to cultivate a supportive climate to maintain workplace equality and develop positive reciprocal relationships between co-workers; they should also be aware of exploitative relationships with their subordinates and take actions to improve open communication and mutual understanding, which facilitate effective vertical social exchange in the future. Simultaneously, employees in organizations should also be concerned about establishing good social networks with their co-workers and avoid engaging in vicious competition. Finally, under different cultural backgrounds, organizations need to adapt to local conditions and timely adjust measures to cope with knowledge-hiding behaviors.

## 8 Limitations and further research directions

The first limitation of this meta-analysis is the small number of primary studies included for each pairwise relationship, which may have influenced the reliability of the meta-analytic findings because of sampling errors. We anticipated this issue in the process of data coding, as there were only 12 years of knowledge-hiding research from which to collect data. As instructed by Lim (2021), the precision of the meta-analytic effect sizes can be enhanced by increasing the number of primary studies included, which indicates the importance of integrating a larger number of studies in future meta-analytic investigations. In addition, the lack of samples within moderator subgroups influenced the accuracy of the moderation analysis, as Q-statistics exhibits poor statistical power in identifying the true heterogeneity with a small number of primary studies (Huedo-Medina et al., 2006; Miao et al., 2016). This can explain why some moderators are insignificant and meaningless regarding certain relationships between knowledge hiding and its antecedents.

Second, this study did not distinguish between studies conducted at individual, team, and organizational levels. Most primary studies included therein were conducted at the individual level, with only a few exceptions that focused on team and organizational levels. Therefore, it is necessary to examine knowledge hiding at higher levels, such as the team and organizational levels. Further investigations to explore the knowledge-hiding phenomenon from a multilevel perspective are thus essential. The multilevel investigation of knowledge hiding has the potential to reveal the “black box” of operative mechanisms of knowledge hiding at higher levels, enhancing the generalizability of previous research findings in the field of knowledge hiding (Huo et al., 2016).

Third, this study developed a nomological framework of knowledge hiding from the social exchange perspective, which theoretically demonstrated the explanatory power and substantial value of SET in the field of knowledge hiding (Anand et al., 2022). However, we should also recognize that SET is not perfect enough by exhibiting a limited predictive validity in explaining some specific relationships between knowledge hiding and its correlates. Given that the conflicting result was found regarding the relationship between knowledge hiding and creativity from the lens of social comparison (Zakariya and Bashir, 2021), we should highlight the deficiency of SET. In essence, SET mainly relies on the benefit–cost analysis to decide whether to engage in or disengage from a social relationship (Ahmad et al., 2023). It seems plausible but oversimplifies the complex dynamics inherent in human relationships to some extents because of disregarding the significance of emotions and subjective initiatives, especially irrational ones (Redmond, 2015). As such, we need to further investigate some new theoretical perspectives to compensate for the limitations of SET in justifying knowledge hiding phenomenon. This entails exploring theories rooted in social cognition or psychology. Some scholars suggested that cognition-related theory provides a more holistic overview of the quality of social exchange by considering the importance of self-efficacy (Liao et al., 2010).”

Finally, the primary studies included in this meta-analysis mainly focused on the composite form of knowledge hiding and ignored the necessity of investigating its specific facets. Some scholars have called for further examination of whether the relationships between knowledge hiding and its correlates vary across the three sub-dimensions: evasive hiding, playing dumb, and rationalized hiding (Wang et al., 2022). It is thus necessary to further examine and compare the predictive validity of these sub-dimensions of knowledge hiding in analyzing certain outcomes when considering their differential levels of deceptiveness.

## Data Availability

The original contributions presented in the study are included in the article/supplementary material, further inquiries can be directed to the corresponding author.
